# Translation of the Child and Adolescent HARDSHIP (Headache-Attributed Restriction, Disability, Social Handicap and Impaired Participation) Questionnaire into the Lithuanian Language and Validation of Its HRQoL (Headache-Related Quality of Life) Scale

**DOI:** 10.3390/ijerph15081579

**Published:** 2018-07-25

**Authors:** Diana Genc, Apolinaras Zaborskis, Nerija Vaičienė-Magistris

**Affiliations:** 1 Faculty of Public Health, Medical Academy, Lithuanian University of Health Sciences, LT44307 Kaunas, Lithuania; 2 Institute of Health Research and Department of Preventive Medicine, Faculty of Public Health, Medical Academy, Lithuanian University of Health Sciences, LT44307 Kaunas, Lithuania; apolinaras.zaborskis@lsmuni.lt; 3 Department of Neurology, Faculty of Medicine, Medical Academy, Lithuanian University of Health Sciences, LT44307 Kaunas, Lithuania; nerija.vaiciene@gmail.com

**Keywords:** headache disorders, burden, questionnaire, translation, children, adolescents, quality of life, Global Campaign against Headache, Lithuania

## Abstract

Recently developed and originally published in English, the Child and Adolescent HARDSHIP (headache-attributed restriction, disability, social handicap and impaired participation) questionnaire is valid and acceptable for the global assessment of the burden of headache in children and adolescents. The present study aimed to translate, adapt and validate a Lithuanian version of this questionnaire. A total of 22 volunteers from 7 to 17 years of age completed the questionnaire with 24 h test-retest and a representative sample of 2505 schoolchildren of the same age participated in the main study. Test-retest reliability of the HRQoL (Headache Related Quality of Life) scale in the translated questionnaire showed substantial agreement (kappa: 0.604). Reliability and validity of the translated HRQoL scale were acceptable (Cronbach’s alpha: 0.749; test-retest kappa: 0.604, test for discriminant validity demonstrated that quality of life decreased by severity of headache). Factorial analysis revealed the two-dimensional structure of the HRQoL scale with indices of good model fit to the collected data. A total of 92.2% of the surveyed children had experienced headache in their lifetime, 74.2% during the last year. Girls and older children experienced headache more often than participants from the other groups. The translated Lithuanian version of the questionnaire seems to be a valid, feasible and acceptable instrument to measure the extent of the burden of headache in large populations.

## 1. Introduction

The global burden of headache is a significant health concern. It has been assessed as high in adults in Europe and worldwide [1,2]. Headaches are also common in childhood and adolescence and can cause significant distress and disability for children and their families [3]. However, data for children and adolescents are sparse [4]. The prevalence of headache disorders in children varies extensively with the occurrence of any type of headache ranging between 33% and 90%, of migraines ranging from 3% to 9.1%, and the prevalence of tension headache ranging from 10% to 24% [5,6,7,8]. This high variability in prevalence may be a result of the different instruments used in studies of children [9]. Information about the burden of headache for Lithuanian children is also limited. One study [10] was performed by using the PedsQLI (pediatric Quality of Life Inventory) to report the impact of headaches on the quality of life of Lithuanian schoolchildren, but there are still no valid measures for children’s headache-attributed restriction, disability, social handicap and impaired participation, and no Headache Related Quality of Life (HRQoL) scale in the Lithuanian language. 

Guidelines for population-based studies of the burden of headache have been developed by the Global Campaign against Headache [11,12]. As a result of this campaign, a questionnaire measuring the headache-attributed restriction, disability, social handicap and impaired participation was developed and named HARDSHIP. The questionnaire has been used and validated in adult studies in multiple countries, languages and cultures [13]. The Child and Adolescent HARDSHIP questionnaires for children aged 6–11 years and adolescents aged 12–17 years were developed by Wöber-Bingöl et al. [4] to assess the global burden of headache in children and adolescents. They can be used to evaluate the prevalence of headache, the impact of headache on quality of life and as a diagnostic tool to differentiate between types of headache. An important part of the questionnaire is the Headache Related Quality of Life (HRQoL) scale. The scale includes questions from KINDL^®^ [14], which seem to be the most appropriate for assessing quality of life [4]. 

The Child and Adolescent HARDSHIP questionnaires, including the survey methodology, were proposed as an acceptable, valid and feasible instrument for a global assessment of the burden of headache in children and adolescents [4]. Questionnaires have already been translated into the Danish language. A Danish pilot study showed high reliability and acceptable validity among young populations and it also indicated that headache may be a major problem for schoolchildren in Denmark [15]. Therefore, the aim of the present study was to translate, adapt and validate a Lithuanian version of the Child and Adolescent HARDSHIP questionnaire. One of the specific goals was to evaluate the psychometric characteristics of the HRQoL scale, which is the major part of the instrument.

## 2. Materials and Methods

The study was conducted as a part of the ongoing epidemiologic study of pediatric headache in Lithuania and the Global Study of Burden of Headache in Children and Adolescents [16]. 

### 2.1. Ethical Approval

The study was approved by Kaunas Regional Committee of Bioethics (BE-2-7, 26-01-2016). Regional educational authorities authorized the study, and school managers and teachers agreed to participate in the survey. Informed consent was obtained from all participants and their parents.

### 2.2. The HARDSHIP Questionnaire and HRQoL Scale

The HARDSHIP questionnaire is a modular instrument incorporating demographic data, diagnostic questions based on ICHD-3 beta criteria and enquiries into components of headache-attributed burden including the HRQoL scale [13]. The questionnaire is designed for application by trained lay interviewers. 

Both Child and Adolescent HARDSHIP questionnaires consist of a total of 44 items: one to record the date; two demographic questions; two screening questions for headache prevalence; 10 headache diagnostic questions based on ICHD-3 beta criteria; four questions enquiring about the frequency of headache and use of abortive medication; four questions related to activity loss; three questions related to headache yesterday; six questions referring to other aspects of headache-attributed burden, such as impact on emotions, self-support, participation; and the 12 questions from the HRQoL scale [4].

In the HRQoL scale, the respondents were asked to indicate the frequency of occurrence of the specified event over the last four weeks. The list of specified events and codes of corresponding items were as presented in Table 1. Response options and scores were: ‘never’ (0); ‘seldom’ (1); ‘often’ (2); and ‘always’ (3). In the analyses, items 1, 2, 5, 6, 7 and 11 were reverse-scored. All scores were subsequently summed, producing a total score. A higher total score indicated a better quality of life.

### 2.3. Translation of the Child and Adolescent HARDSHIP Questionnaires

The translation was performed in accordance with the guidelines proposed by the Translation Working Group for the translation of documents produced by the Lifting the Burden group [17]. The principles of good practice proposed by International Society For Pharmacoeconomics And Outcomes Research were also taken into consideration [18].

Forward translations were prepared by two independent bilingual Lithuanian residents and sent to the translation coordinator. Discrepancies in the forward translations were discussed with translators until a consensus on final forward translation was reached. Issues of reconciliation were documented.

One back-translation of the consensus-based forward translation was carried out by a professional translator. The back-translation was forwarded to the original author with a request to compare the original and back-translated versions of the questionnaires and to assess their conceptual equivalence.

The harmonization was done by the forward translators, and a language competent person (elementary school teacher with Lithuanian as mother tongue) with a translation coordinator as the chairperson. Target audience review (face validation) was carried out by an investigator who interviewed six children affected by headache disorders within the target ages of 7–17 years. Children were asked to verbalize their thoughts and opinions while filling out their questionnaire. The results of the audience review were considered and a final translated version of each questionnaire was developed.

The target audience review did not prompt major changes in adolescent the HARDSHIP questionnaire. The face validation, however, showed that younger children experienced difficulties in understanding international words or words with double meanings; therefore, minor changes were implemented in the Child HARDSHIP questionnaire by choosing synonyms. The Child HARDSHIP questionnaire is simpler and more comprehensible for children below 12 years of age but the exact meaning of the Adolescent HARDSHIP questionnaire is kept. It is worth mentioning that some children had difficulty in answering ‘yes’ or ‘no’ and they preferred to have the choice of ‘sometimes’ for headache diagnostic questions. 

The final translated versions were proofread and checked for spelling and grammar errors. The layout was finalized by an expert committee consisting of the project investigators and a teacher. A final report about the translation procedure was accepted by the developers of the questionnaires.

### 2.4. Data Collection

A test-retest study was organized in one school. Two children (a girl and a boy) from eleven classes (total 22 children) within the target age of 7–17 years were asked to volunteer to fill out questionnaires twice in the period of 24 h. The study sample was randomly heterogeneous in respect to headaches. This study confirmed the feasibility of the methodology with only minor modification of questionnaire wording and confirmed the organization of data collection procedures.

As the final aim of the global study is to calculate headache prevalence and the burden, we followed the requirements of the protocol to collect at least 200 evaluable participants in each age group. In Lithuania, the main study was undertaken in 2016 in 23 randomly selected schools located in seven different regions of Lithuania, thus the socio-economic diversity of the country was well represented. All classes in each school across the age ranges 7–11 years and/or 12–17 years were invited to participate in the study. All children in the class were invited to complete the questionnaires. A total initial sample consisted of 4019 invited children.

**Figure 1:**
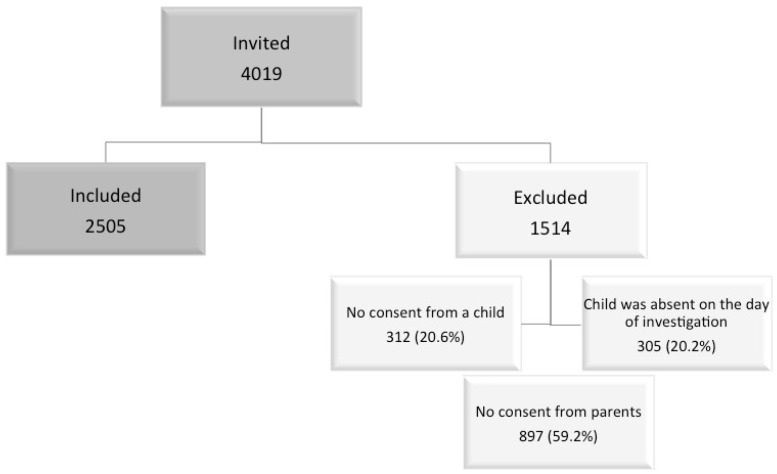
Flow diagram of the data collection process.

Two days before the study start, an informed consent form for schoolchildren and their parents was distributed. On the day of the study, children who agreed and possessed an informed consent form signed by their parents were invited to complete the questionnaire. They completed an anonymous questionnaire during one class under the supervision of an investigator, or an instructed school public health specialist or/and a teacher. After an initial introduction, schoolchildren able to read completed the questionnaires independently. Children in the 7–8 year age group and a few older children with learning difficulties were assisted. The flow diagram of the data collection is presented in Figure 1.

### 2.5. Statistical Analysis

The data were analyzed using the SPSS (Version 21.0; SPSS Inc, Chicago, IL, USA, 2012) statistical package supplemented with AMOS (Analysis of Moment Structures) [19].

Descriptive statistics were employed to calculate the means, medians and standard deviations of the continuous variables, as well as to calculate percentages of the categorical data. To evaluate potential differences between respondent groups in all categorical variables, we applied a z-test or a Chi-square test. The differences between respondent groups in the summed scores of the scale were tested with nonparametric Mann-Whitney U test (2 groups) or Kruskal-Wallis test (more than 2 groups). The cut-off level for statistical significance was set at 0.05. 

In order to understand the interrelations among the measures and to confirm the inherent structure of the scale, an explanatory factor analysis (EFA) was performed. Using the SPSS factor analysis procedure, we carried out a Principal Component Factor analysis with a Promax rotation since factors were correlated. The appropriateness of the models was evaluated with the Kaiser-Meyer-Olkin (KMO) measure along with the Bartlett’s test (KMO ≥ 0.5 and *p* < 0.001 show the adequacy of the data for use in the EFA). Factors were extracted based on the break point of successive eigenvalues (≥1), item factor loadings (≥0.4) and interpret ability. 

Confirmatory factor analysis (CFA) was employed to confirm construct validity [19,20]. The goodness of fit of the models was evaluated using the following measures on goodness-of-fit: Comparative Fit Index (CFI), Tucker-Lewis Index (TLI) and the Root Mean Square Error of Approximation (RMSEA) that was considered as the main criterion. CFI and TLI values close to 1 (≥0.90), and RMSEA values close to 0 (≤0.09) indicate a good fit [20]. The CFA was realized with AMOS software (Version 21.0; SPSS Inc, Chicago, IL, USA, 2012) [19].

Regarding the generalizability of the findings, factor analysis was performed by randomly splitting the data set into two groups [21]. This meant that participants from each school were assigned to group 1 when their subject number was an odd number (N = 1253) or to group 2 when their subject number was an even number (N = 1252). Statistical comparisons between the groups on their demographic and clinical characteristics, and mean item scores for the HRQoL scale was conducted; there were no statistical differences between groups. Subsequently, EFA was conducted in group 1 and CFA was conducted in group 2.

A set of tests was used for the examination of the psychometric properties of the HRQoL [22,23,24]. The Cronbach α was used as a measure of internal consistency of the total scale. A Cronbach *α* ≥ 0.70 was considered acceptable. Furthermore, other tests of internal reliability (inter-item and item-total correlations) were also investigated.

Test-retest reliability was assessed by using the Spearmen correlation coefficient, the kappa statistic and intraclass correlation coefficient (ICC) using a two-way random effects model for the HRQoL score.

## 3. Results

### 3.1. Sample Characteristics

A total of 2505 schoolchildren between 7 and 17 years (Mean ± SD: 11.51 ± 3.17), of which 1382 (55.2%) were 7–11 years old and 1169 (46.7%) were boys, submitted qualitatively completed questionnaires that were included in the present analysis. The response rate of the study was 62.3%. The group of non-participants did not differ significantly from the group of participants in regard to their distribution by gender and age group.

A total of 92.2% of the surveyed children had experienced headache in their lifetime, 74.2% during the last year. Girls and older children experienced headache more often than participants from the other groups. A total of 59.0% had experienced headaches during the last week at least once and almost half (47.7%) of them, or 28.3% of the entire sample, had taken pills or medication due to headache. Children who reported headache in the past year were analyzed for the duration and severity of their headache: gender and age were important factors for those headache characteristics (Table 2).

### 3.2. Descriptive Statistics of the HRQoL Scale

There was a good overall response rate to the items on the scale (in fact, 8 items were left blank in the total dataset of the scale, no child missed more than one item; missing data were corrected with an individual average value). Table 3 shows the distribution of scores by HRQoL items. Notable floor effects were detected for negative items (e.g., ‘1 Ill’), while ceiling effects were noticed for several positive items (‘9 Fine at home’ and ’10 Friendship’). It was unlikely that the floor or ceiling effects were more prominent for the items that were reverse-coded.

The HRQoL summed score was found to be skewed (skewness = −0.495) and not normally distributed. Its values ranged from 0 to 36, with a mean of 24.23 (a median of 25) and a standard deviation of 5.13 (Table 4). It was higher (i.e., HRQoL was better) for boys than for girls (24.86 ± 4.95 vs. 23.68 ± 5.23, *p* < 0.001), and for younger than for older respondents (24.49 ± 5.36 vs. 23.91 ± 4.83, *p* = 0.001). These results indicate that the measure detected substantial variability in the value of HRQoL. 

### 3.3. Internal Consistency

Cronbach’s alpha resulted in 0.749 for the total HRQoL scale, indicating an acceptable internal consistency. 

### 3.4. Discriminant Validity

The discriminant validity of the scale was tested by assessing HRQoL summed scores for self-reported headache occurrence in the last year, its average duration and severity (Table 5). The children who reported at least one headache in the last year provided significantly lower scores for the scale in comparison with children who did not experience headache in the period (a reference group). In parallel with this, a significant negative gradient of summed scores was observed across the groups of respondents by average duration of headache episodes and their severity. Multiple comparisons (post hoc LSD (Fisher’s Least Significant Difference) test) showed a significant difference between all groups of children by average duration of headache episodes except between ‘about 2–4 h’ and ‘more than 4 h’ groups (*p* = 0.239), and a significant difference between all groups of children by severity of headache onset. Therefore, a predicted hypothesis concerning discriminant validity of the HRQoL scale was confirmed. 

### 3.5. Test-Retest Reliability

Twenty-two children (mean age ± SD: 12.00 ± 3.24; 50% boys) completed the questionnaire twice at a 24 h interval. They did not report any change in their perceived health status between testing points. No significant change in mean scores of HRQoL between the first and second measurements was observed, indicating that there was no upward or downward shift in the values. As further evidence of test-retest reliability, the total scores correlated extremely well (ρ = 0.968), the kappa statistic was 0.604 and the ICC was 0.984 (95% CI: 0.962–0.993). 

### 3.6. Factor Analysis

The explanatory factor analysis (EFA) of the study sample revealed a two-factor solution of the scale, which accounted for 40.1% of total variance. Table 6 presents the factor structure of the HRQoL scale (the result of the Principal Component Analysis with Promax rotation is reported). Factorial analysis on sub-samples of boys and girls supported the two-factor solution and confirmed the same factor structure (results not presented). The first component combined six items that had excellent loadings of the first component but relatively small loadings of the second component. It accounted for 26.4% of total variance. The maximal loading (0.785) was found for item ‘8 Pleased’ and the remaining items also represented positive life aspects, which indicates a higher value of life quality when they occur frequently. The second component combined the remaining six items and was clearly distinguished by large loadings of the second component. It accounted for 13.7% of total variance. All items of this component represented negative life aspects, which decreases the HRQoL if they occur frequently. The Spearman correlation coefficient between components was 0.298 (*p* < 0.01). There was a strong correlation between the total score and component 1 (ρ = 0.861), as well as between the total score and component 2 (ρ = 0.706); both correlations were significant at *p* < 0.01.

**Figure 2:**
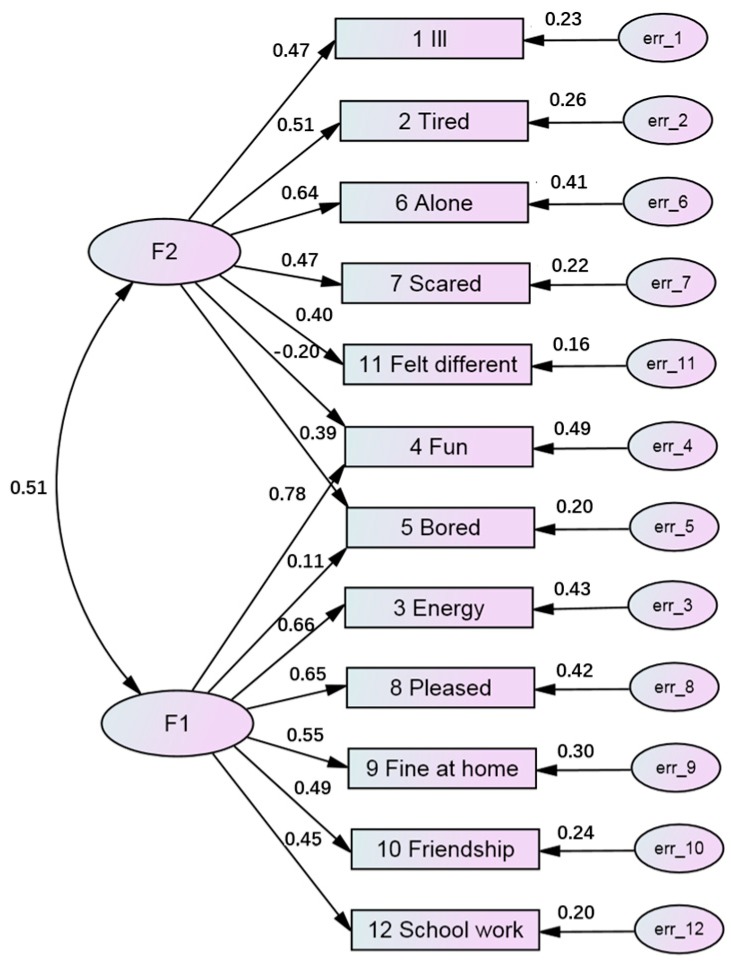
Path diagram of confirmatory factor analysis with standardized estimates (N = 1252). The left ellipses represent factors F1 and F2, the right ellipses (err_1, err_2, etc.) represent measurement errors of corresponding input variables (1 Ill, 2 Tired, etc.). The numbers on the left one-sided arrows (0.47, 0.51, etc.) are estimates of factor weights and 0.51 is the correlation between F1 and F2. The numbers on the right arrows (0.23, 0.26, etc.) are the squared multiple correlations, which indicate a proportion of variable variance accounted for by factor F1 and/or factor F2.

The dimensionality of the HRQoL scale was confirmed by the CFA. Based on the above EFA, we hypothesized that CFA would show two factors (latent variables). In addition, we postulated that these factors may be correlated, because each of them has a connection with a limited number of scale items (in contrast to EFA, factors are connected with all scale items). Figure 2 demonstrates a path diagram of CFA with standardized estimates (N = 1253), which was constructed on the basis of randomly selected sub-group 2 of the entire sample. All factor loadings (the path coefficients leading from the common factors to the observed variables) were found to be significant. It can also be seen that items ‘4 Fun’ and ‘5 Bored’ were associated with both positive and negative dimensions of the scale. The factors were moderately correlated (*r* = 0.509). Indices of good fit were assessed. A RMSEA score (0.055, 90% CI: 0.048–0.062) indicates a high model fit in the total sample, and other fit indices, namely TLI and CFI, also suggest sufficient model fit in the entire data set (Table 7).

In order to compare the factor structure of HRQoL between groups of participants who reported headache or no headache in the last year, we ran CFA and tested whether both groups of participants followed the same factor structure (Table 7). These analyses confirmed the a priori expectations. Moreover, they revealed an essential improvement in model fit in the group of participants who reported no headache in the past year (RMSEA: 0.030, 90% CI: 0.016–0.042; TLI: 0.956; CFI: 0.966). In contrast, model fit was not as good among participants who reported headache in the last year (RMSEA: 0.070, 90% CI: 0.064–0.075; TLI: 0.852; CFI: 0.886). However, the model for this group can be improved. Analysis of modification indices showed a substantial gain in statistics of the model fit to survey data if correlations between errors of variables 1 Ill and 2 Tired, 2 Tired and 3 Energy, 5 Bored and 7 Scared were allowed. The estimated correlations were significant at *p* < 0.001 and were equal to 0.23, −0.20 and −0.19, respectively. Then, the model fit was acceptable (RMSEA: 0.057, 90% CI: 0.051–0.062; TLI: 0.903; CFI: 0.930).

## 4. Discussion

Studies on the global burden of headache in children and adolescents provide fundamental information about headache disorders worldwide [4]. However, rigorous studies are required to quantify this burden and must use measures that are valid and reliable. The development of such measure should be informed by both theoretical and practical perspectives [9]. Recently developed and originally published in English, the Child and Adolescent HARDSHIP questionnaires collect comprehensive information that is valid, feasible and acceptable for a global assessment of the burden of headache in children and adolescents [4]. This study aimed to translate, adapt and validate a Lithuanian version of the Child and Adolescent HARDSHIP questionnaires in order to assess the extent of burden of headache among Lithuanian children and adolescents as well as evaluate the psychometric characteristics of the HRQoL scale, which is a major part of the questionnaire. 

### 4.1. Lithuanian Version of the Child and Adolescent HARDSHIP Questionnaires

No major problems were experienced in producing forward translations of the questionnaires. A translation of HARDSHIP questionnaires and their cultural adaptation procedure was also done in Denmark [15]. In that study as well as in our study, the face-to-face interviews showed that pupils in the age group of 7–11 years had some difficulty in understanding a few of the questions. 

There was a good overall response rate to the questions in the questionnaires. For example, only 8 items were left blank in the total dataset for the HRQoL scale. As there was no specific pattern in the non-responses, we concluded that the low number of unanswered questions would not affect the acceptability of the translated questionnaires.

### 4.2. Psychometric Characteristics of the HRQoL Scale

In this study, we evaluated the psychometric properties of the HRQoL scale, which is a major part of the HARDSHIP questionnaire. This is the first time that such a thorough evaluation of the psychometric properties of this scale, in particular its factor analysis, was performed for the HARDSHIP questionnaire. For this reason, we do not have other study outcomes with which we can compare the results of our research. 

The central finding of this analysis was the demonstration of satisfactory reliability and validity for the HRQoL scale which was translated into Lithuanian language and culturally adapted for use among Lithuanian children and adolescents. Cronbach’s alpha coefficient for the total scale revealed adequate homogeneity of the 12 items in the measure of HRQoL. Many methodologists recommend a minimum alpha coefficient between 0.65 and 0.8 (or higher in many cases), thus the obtained value (0.749) could be considered acceptable [24]. 

Discriminant validity of the scale was determined by comparing its scores between the clinical groups. Statistically significant results were obtained between all groups of respondents by severity of headache. This finding is in line with the results of the study of Wöber-Bingöl et al. [4] who reported that quality of life was poorer in respondents with headache than in those without. Therefore, discriminant validity of the HRQoL scale was confirmed. In this regard, the scale performs well as a discriminant measure. However, since the scale is intended to be used as an outcome measure in clinical trials, its evaluative properties need to be assessed. Therefore, longitudinal studies are necessary to determine its minimal clinical importance [25,26]. The HRQoL scale, as well as the entire Child and Adolescent HARDSHIP questionnaire, is intended to measure the burden of headache in large populations and there is, therefore, no need to reflect the condition of individuals [15].

The test-retest study demonstrated substantial agreement between test and retest in the items of the HRQoL scale. Although longer test-retest timing is recommended, the 24 h period was chosen with respect to questions about headache yesterday and last week. A longer time interval could misrepresent results of headache frequency whereas shorter recall intervals may improve accuracy [27]. Although shorter test-retest timing may be associated with the recollection of answers [28], we considered the difficulty of recalling answers to specific questions in the questionnaire which includes 44 questions. 

The strength of the validation of the HRQoL scale in our study is the use of factor analysis techniques. We employed the EFA for checking the dimensionality of the scale. This analysis showed that the scale has two dimensions. The first combines positive events with the quality of life, while the second combines negative events with the quality of life. At first glance, it seems that such division of the items into dimensions could be related to the inappropriate scoring of items (during the analysis, all negative events were reverse-scored). However, this assumption was negated by the CFA. The CFA analysis, a specific method of Structural Equation Modeling (SEM), is a second-generation multivariate method that is used to assess the reliability and validity of the model measures [19,20,21]. All items of the scale were found to be significant in the revealed “positive-negative” dimension structure of the HRQoL scale. The model fit to the survey data in randomly selected sub-groups of participants was high, which allows the study findings to be generalized [21]. In addition, this analysis showed that the model is appropriate for both groups of participants, those who had not reported and those who had reported headache in the last year. However, for the second group of respondents (who had reported headache in the last year) the relationships between several items of the scale were well-defined. For instance, being ill was associated with being tired and, consequently, with having lower energy. We have no clear explanation for this phenomenon, but it does not limit the application of the HRQoL scale for population-based studies of the burden of headache. 

### 4.3. The Extent of Burden of Headache in Schoolchildren in Lithuania

The study demonstrates that headache is a major problem among children and adolescents in Lithuania and that it has a significant effect on their quality of life. Our study showed high prevalence of headache among schoolchildren (92.2% per lifetime, 74.2% per 1-year period and 59% per 1-week period). A pilot study performed in Austria and Turkey [4] and a nationwide cross-sectional survey conducted in Turkey [29] using HARDSHIP questionnaires disclosed similar findings. They found that the crude 1-year prevalence of headache in the whole sample was 89.3% in the pilot study and 73.7% in the nationwide survey in Turkey. The prevalence observed in our study seems very high compared with previously published data, for example, 58.4% (95% CI 58.1–58.8) in a study of Abu-Arafeh et al. in 2010 [5] or 54.4% (95% CI 43.1–65.8) as per Wöber-Bingöl in 2013 [7]. It is difficult to compare our findings with other data due to the different instruments and methodology used. However, strict study protocol and the large sample size means we can be confident in the results of our study. As this study was conducted as part of the ongoing Global Study of Burden of Headache in Children and Adolescents [16] further analysis of the data and results observed in other countries across different continents will allow for comparing the prevalence and the burden of headache in schoolchildren across the world.

### 4.4. Limitations

Conceptual limitations of the HARDSHIP questionnaire have already been described by the developers of the questionnaire [4] and other authors [15]. Our study has some specific limitations. Firstly, we worked on the full HARDSHIP questionnaire although the reliability and validity tests were only employed for the HRQoL scale. This was mainly related to the fact that only this scale had a typical scale construct asking children to respond to a series of statements about headache related quality of life in terms of occurrence of disorders or satisfactions, while the remaining items refer to a variety of aspects of headache-attributed burden (e.g., headache prevalence, diagnosis and medication). As test-retest of this scale demonstrated substantial agreement in quality of life questions, it is likely that similar results would be obtained by testing with burden related HARDSHIP questions. Secondly, in this study we did not test how the HRQoL is associated with similar scales, and due to this limitation, we were unable to estimate the construct validity of the scale. As an example of such a scale, the WHO-5 Well-being Index, which is freely available in several languages could be chosen [30]. It has been shown to be a reliable measure of emotional functioning and a good screener for mental disorders and pain in pediatrics care [31]. We could not add the WHO-5, or other similar scales, to the questionnaire as this would distort the originality of the HARDSHIP questionnaire. Thirdly, our study did not estimate the time needed to complete the questionnaire. Based on other study data that showed that the questionnaire can be completed in a short time (younger children fill the questionnaire within 30 min) [4,15], we considered that the time required to fill our questionnaire was not a major issue.

## 5. Conclusions

The newly translated Lithuanian version of the Child and Adolescent HARDSHIP questionnaire seems to be a valid, feasible and acceptable instrument that complies with the intentions of the initiators of the study aiming to measure the extent of the burden of headache in large populations. The study also indicates that headache is a major problem among children and adolescents in Lithuania that diminishes their quality of life. These findings clearly demonstrate the need to further investigate the burden of headache among Lithuanian schoolchildren. 

## Figures and Tables

**Table 1:** Items from the HRQoL scale.

Item Code	Specified Event
1 Ill	I felt ill
2 Tired	I was tired and worn-out
3 Energy	I felt full of energy
4 Fun	I had fun and laughed a lot
5 Bored	I was bored
6 Alone	I felt alone
7 Scared	I was scared
8 Pleased	I felt pleased with myself
9 Fine at home	I felt fine at home
10 Friendship	I got along with my friends
11 Felt different	I felt different from other children
12 School work	Doing my schoolwork was easy

HRQoL: Headache Related Quality of Life.

**Table 2:** Headache characteristics in percent, by gender and age.

Characteristics		Gender			Age Group			Total Sample (N = 2505)
		Boys (*n* = 1169)	Girls (*n* = 1336)	*p*-Value ^a^	7–11 (*n* = 1382)	12–17 (*n* = 1123)	*p*-Value ^a^	
Headache in life		90.8	93.4	0.014	88.6	96.6	<0.001	92.2
Headache in the last year		67.8	79.7	<0.001	62.7	88.3	<0.001	74.2
Headache in the last week		50.3	65.5	<0.001	54.6	62.9	<0.001	59.0
Have taken pills or medication due to headache		22.7	32.4	<0.001	24.8	31.2	0.002	28.3
Duration of headache	no pain	32.2	20.3	<0.001	37.3	11.7	<0.001	25.8
	less than hour	37.4	40.0	0.182	39.9	37.3	0.183	38.8
	1-2 h	17.4	25.5	<0.001	13.8	31.4	<0.001	21.7
	2.1-4 h	8.4	9.1	0.536	5.3	13.0	<0.001	8.7
	more than 4 h	4.7	5.2	0.569	3.6	6.6	0.001	5.0
	*p*-value ^b^	<0.001				<0.001		
Severity of headache	no pain	32.2	20.3	<0.001	37.3	11.7	<0.001	25.8
	not bad	33.7	33.0	0.711	28.7	39.1	<0.001	33.4
	quite bad	30.8	41.8	<0.001	29.7	45.2	<0.001	36.6
	very bad	3.3	4.9	0.043	4.3	4.0	0.708	4.2
	*p*-value ^b^	<0.001				<0.001		

^a^ z test to compare proportions in groups (boys vs girls and 7–11 vs. 12–17 years of age). ^b^ Chi square test to compare overall distributions of characteristics in groups (boys vs girls and 7–11 vs. 12–17 years of age).

**Table 3:** Distribution (%) of scores in response to items of the HRQoL scale.

Item	Never (0)	Seldom (1)	Often (2)	Always (3)	Distribution Asymmetry ^a^ (%)	Corrected Item-Total Correlation
1 Ill ^b^	32.6	56.8	8.9	1.6	−39.4	0.312
2 Tired ^b^	17.8	52.6	24.4	5.1	−20.4	0.342
3 Energy	12.7	34.0	36.4	17.0	+3.3	0.482
4 Fun	9.5	26.7	40.1	23.7	+13.8	0.448
5 Bored ^b^	28.0	56.9	11.5	3.6	−34.9	0.385
6 Alone ^b^	56.6	31.7	8.0	3.7	−38.3	0.417
7 Scared ^b^	58.8	34.4	4.9	1.9	−40.5	0.284
8 Pleased	12.7	33.3	33.7	20.3	+4.0	0.500
9 Fine at home	3.9	12.6	24.4	59.2	+33.5	0.413
10 Friendship	2.5	13.6	36.2	47.7	+33.9	0.391
11 Felt different ^b^	39.9	37.6	13.1	9.3	−27.5	0.242
12 School work	12.9	43.0	30.0	14.1	−5.9	0.377

^a^ “−“ represents a floor effect, and “+” represents a ceiling effect, calculated as 50% (the proportion of both “never” and “seldom”). ^b^ indicates that in analyses, the item is reverse-scored.

**Table 4:** Descriptive characteristics of HRQoL summed scores by groups of respondents.

Group	*n*	Mean	SD	Median	*p*-Value *
Total	2505	24.23	5.13	25	
Boys	1169	24.86	4.95	25	<0.001
Girls	1336	23.68	5.23	24	
7–11 years	1382	24.49	5.36	25	0.001
12–17 years	1123	23.91	4.83	24	

* *p*-values obtained from Mann-Whitney U test.

**Table 5:** Discriminant validity of the HRQoL scale.

Occurrence and Character of Headache		*n*	Mean	SD	Median	*p*-Value ^a^
Headache	in the last year	1858	23.68	4.68	24	<0.001
	no headache	647	25.82	5.17	26	
Duration	more than 4 h	124	21.58	5.89	22	<0.001
	about 2–4 h	219	22.24	5.22	22	<0.001
	about 1 h	544	23.02	5.06	23	<0.001
	about half hour	971	24.63	4.92	25	<0.001
	no headache	647	25.82	4.68	26	-
Severity	very bad	647	21.16	6.14	21	<0.001
	quite bad	918	22.59	5.19	23	<0.001
	not bad	835	25.19	4.56	26	0.006
	no headache	647	25.82	5.17	26	-

^a^*p*-values obtained from Mann-Whitney U test comparing with ‘no headache’ as a reference group.

**Table 6:** Factor loadings in the explanatory factor analysis ^a^ of the HRQoL scale (N = 1253).

Item ^b^	Component ^c^	
	1	2
8 Pleased	**0.785**	−0.036
4 Fun	**0.763**	−0.101
3 Energy	**0.734**	−0.040
9 Fine at home	**0.588**	0.026
10 Friendship	**0.532**	0.018
12 School work	**0.477**	0.136
6 Alone	0.040	**0.672**
2 Tired	0.011	**0.653**
1 Ill	−0.039	**0.606**
7 Scared	−0.060	**0.570**
11 Felt different	−0.080	**0.556**
5 Bored	0.245	**0.481**

^a^ Extraction method: Principal Component Analysis; rotation method, Promax with Kaizer Normalization, rotation converged in 3 iterations. ^b^ Items are sorted by loadings. ^c^ The bolded terms indicate the main loadings for corresponding dimensions.

**Table 7:** Estimates of two-factor model of the HRQoL scale obtained from the confirmatory factor analysis of randomly selected sub-group 2 of the entire sample and sub-samples of participants who reported headache or no headache in the last year.

	Estimates		
	Randomly Selected Sub-group of the Entire Sample (N = 1252)	No Headache in the Last Year (N = 647)	Headache in the Last Year (N = 1858)
Standardized regression weights (factor loadings) and direction of associations:			
1 Ill 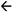 F2	0.474	0.355	0.493
2 Tired 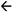 F2	0.513	0.392	0.552
6 Alone 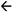 F2	0.642	0.508	0.632
7 Scared 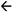 F2	0.471	0.510	0.447
11 Felt different 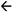 F2	0.401	0.406	0.391
4 Fun 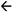 F2	–0.200	–0.080	–0.152
4 Fun 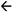 F1	0.783	0.592	0.771
5 Bored 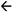 F2	0.387	0.329	0.403
5 Bored 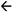 F1	0.107	0.158	0.153
3 Energy 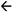 F1	0.658	0.543	0.681
8 Pleased 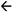 F1	0.652	0.616	0.697
9 Fine at home 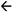 F1	0.549	0.523	0.511
10 Friendship 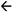 F1	0.492	0.553	0.439
12 School work 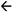 F1	0.448	0.420	0.425
Correlations			
F1 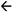 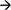 F2	0.509	0.384	0.445
Model fit estimates			
Chi-squared	246.2	80.0	513.7
DF	51	51	51
Chi-squared/DF	4.828	1.569	10.072
*p*-value	<0.001	-	< 0.001
TLI	0.901	0.956	0.852
CFI	0.924	0.966	0.886
RAMSEA (90% CI)	0.055 (0.048–0.062)	0.030 (0.016–0.042)	0.070 (0.064–0.075)

## Abbreviations

HRQoL	Quality of Life
PedsQLI	Pediatric Quality of Life Inventory
HARDSHIP	Headache-attributed restriction, disability, social handicap and impaired participation
SPSS	The Statistical Package for the Social Sciences
EFA	Explanatory Factorial Analysis
KMO	Kaiser-Meyer-Olkin
CFA	Confirmatory factor analysis
CFI	Comparative Fit Index
TLI	Tucker–Lewis Index
RMSEA	Root Mean Square Error of Approximation
ICC	Intraclass Correlation Coefficient
SEM	Structural Equation Modeling
WHO-5	World Health Organization Well-Being Index

## References

[1] Steiner T.J., Stovner L.J., Katsarava Z., Lainez J.M., Lampl C., Lantéri-Minet M., Rastenyte D., Ruiz de la Torre E., Tassorelli C., Barré J. (2014). The impact of headache in Europe: Principal results of the Eurolight project. J. Headache Pain.

[2] Vos T., Abajobir A.A., Abbafati C., Abbas K.M., Abate K.H., Abd-Allah F. (2017). Global, regional, and national incidence, prevalence, and years lived with disability for 328 diseases and injuries for 195 countries, 1990–2016: A systematic analysis for the global burden of disease study 2016. Lancet.

[3] Blume H.K. (2017). Childhood Headache: A Brief Review. Pediatr. Ann..

[4] Wöber-Bingöl Ç., Wöber C., Uluduz D., Uygunoğlu U., Aslan T.S., Kernmayer M., Zesch H.E., Gerges N.T.A., Wagner G., Siva. A. (2014). The global burden of headache in children and adolescents—Developing a questionnaire and methodology for a global study. J. Headache Pain.

[5] Abu-Arafeh I., Razak S., Sivaraman B., Graham C. (2010). Prevalence of headache and migraine in children and adolescents: A systematic review of population-based studies. Dev. Med. Child. Neurol..

[6] Antonaci F., Voiticovschi-Iosob C., Di Stefano A.L., Galli F., Ozge A., Balottin U. (2014). The evolution of headache from childhood to adulthood: A review of the literature. J. Headache Pain.

[7] Wöber-Bingöl Ç. (2013). Epidemiology of migraine and headache in children and adolescents. Curr. Pain.

[8] Straube A., Heinen F., Ebinger F., von Kries R. (2013). Headache in school children: Prevalence and risk factors. Dtsch. Arztebl. Int..

[9] Kernick D., Reinhold D., Campbell J.L. (2009). Impact of headache on young people in a school population. Br. J. Gen. Pract..

[10] Januškevičienė A., Vaitkaitienė E., Zaborskis A. (2013). Headache in school age children, comorbid fatigue and quality of life. Vaikų Pulmonologija ir Alergologija.

[11] Stovner L.J., Al Jumah M., Birbeck G.L., Gururaj G., Jensen R., Katsarava Z., Queiroz L.P., Scher A.I., Tekle-Haimanot R., Wang S.J. (2014). The methodology of population surveys of headache prevalence, burden and cost: Principles and recommendations from the Global Campaign against Headache. J. Headache Pain.

[12] Steiner T.J. (2004). World Headache Alliance: Lifting the burden: The global campaign against headache. Lancet. Neurol..

[13] Steiner T.J., Gururaj G., Andrée C., Katsarava Z., Ayzenberg I., Yu S.Y., Al Jumah M., Tekle-Haimanot R., Birbeck G.L., Herekar A. (2014). Diagnosis, prevalence estimation and burden measurement in population surveys of headache: Presenting the HARDSHIP questionnaire. J. Headache Pain.

[14] KINDL^®^ Revised Questionnaire to Assess Health-Related Quality of Life in Children and Adolescents. http://kindl.org/english/.

[15] Jorgensen J.E., McGirr K.A., Korsgaard H.O., Rathleff M.S. (2016). Translation and validation of the Child and the Adolescent HARDSHIP (Headache-atributed restriction, disability, social handicap and impaired participation) questionnaire into Danish language. Peer J..

[16] Lifting the Burden The Global Campaign Against Headache. http://www.l-t-b.org/index.cfm/spKey/people.child_adolescent_burden.html.

[17] Lifting the Burden The Global Campaign Against Headache. Translation Protocols. http://www.l-t-b.org/index.cfm/spKey/people.translation_protocols.html.

[18] Wild D., Grove A., Martin M., Eremenco S., McElroy S., Verjee-Lorenz A., Erikson P. (2005). Principles of good practice for the translation and cultural adaption process for patient-reported outcomes (PRO) measures: Report of the ISPOR task force for translation and cultural adaption. Value Health.

[19] Arbuckle J.L. (2012). IBM SPSS AMOS 21 User’s Guide.

[20] Albright J.J. Confirmatory Factor Analysis Using AMOS, LISREL and MPLUS. The Trustees of Indiana University 2006–2008. http://www.iu.edu/~statmath/stat/all/cfa/cfa2008.pdf.

[21] Schmitt T.A. (2011). Current methodological considerations in exploratory and confirmatory factor analysis. J. Psychoeduc. Ass..

[22] Streiner D., Norman G. (2000). Health Measurement Scales. A practical Guide to their Development and Use.

[23] Whiston S.C. (2009). Principles and Applications of Assessment in Counselling.

[24] Aldridge V. (2015). Reliability Assessment Using SPSS. University of York, Centre for Applied Statistics Courses, UCL Institute of Child Health. http://www.spssusers.co.uk/Events/2015/ALDRIDGE2015.pdf.

[25] Rothman M.L., Beltran P., Cappelleri J.C., Lipscomb J., Teschendorf B., the Mayo/FDA Patient-Reported Outcomes Consensus Meeting Group (2007). Patient-Reported Outcomes: Conceptual Issues. Value Health.

[26] Guyatt G., Fenny D., Patrick D. (1991). Issues in quality-of-life measurement in clinical trials. Control Clin. Trial..

[27] Houle T.T., Turner D.P., Houle T.A., Smitherman T.A., Martin V., Penzien D.B., Lipton R.B. (2013). Rounding behavior in the reporting of headache frequency complicates headache chronification research. Headache.

[28] Nunnally J.C., Bernstein I.H. (1994). Psychometric Theory.

[29] Wöber C., Wöber-Bingöl Ç., Uluduz D., Aslan T.S., Uygunoglu U., Tüfekçi A., Alp S.I., Duman T., Sürgün F., Emir G.K. (2018). Undifferentiated headache: Broadening the approach to headache in children and adolescents, with supporting evidence from a nationwide school-based cross-sectional survey in Turkey. J. Headache Pain.

[30] The WHO-5 Website. https://www.psykiatri-regionh.dk/who-5/who-5-questionnaires/Pages/default.aspx.

[31] Allgaier A.K., Pietsch K., Frühe B., Prast E., Sigl-Glöckner J., Schulte-Körne G. (2012). Depression in pediatric care: is the WHO-Five Well-Being Index a valid screening instrument for children and adolescents?. Gen. Hosp. Psychiatry.

